# Genome-Wide Introgression and Quantitative Trait Locus Mapping Reveals the Potential of Asian Cotton (*Gossypium arboreum*) in Improving Upland Cotton (*Gossypium hirsutum*)

**DOI:** 10.3389/fpls.2021.719371

**Published:** 2021-08-02

**Authors:** Liuchun Feng, Yu Chen, Min Xu, Ying Yang, Haoran Yue, Qiao Su, Chenhui Zhou, Guoli Feng, Nijiang Ai, Ningshan Wang, Baoliang Zhou

**Affiliations:** ^1^State Key Laboratory of Crop Genetics & Germplasm Enhancement, Jiangsu Collaborative Innovation Center for Modern Crop Production, Nanjing Agricultural University, Nanjing, China; ^2^Shihezi Agricultural Science Research Institute, Shihezi, China

**Keywords:** genome wide introgression, inter-ploidy gene transference, chromosomal structure variation, quantitative trait locus mapping, *Gossypium arboreum*, *Gossypium hirsutum*

## Abstract

*Gossypium arboreum* (2*n*=2*x*=26, A_2_), the putative progenitor of the A_t_-subgenome of *Gossypium hirsutum* (2*n*=4*x*=52, AD), is a repository of genes of interesting that have been eliminated during evolution/domestication of *G. hirsutum*. However, its valuable genes remain untapped so far due to species isolation. Here, using a synthetic amphiploid (AADDA_2_A_2_) previously reported, we developed a set of 289 *G. arboreum* chromosome segment introgression lines (ILs) in *G. hirsutum* by expanding the backcrossing population and through precise marker-assisted selection (MAS) although complex chromosomal structural variations existed between parents which severely hindered introgression. Our results showed the total coverage length of introgressed segments was 1,116.29 Mb, representing 78.48% of the A_t_-subgenome in the *G. hirsutum* background, with an average segment-length of 8.69 Mb. A total of 81 co- quantitative trait loci (QTLs) for yield and fiber quality were identified by both the RSTEP-ADD-based QTL mapping and the genome-wide association study (GWAS) analysis, with 1.01–24.78% of the phenotypic variance explained. Most QTLs for boll traits showed negative additive effects, but *G. arboreum* still has the potential to improve boll-number traits in *G. hirsutum*. Most QTLs for fiber quality showed negative additive effects, implying these QTLs were domesticated in *G. hirsutum* compared with *G. arboreum* and, a small quantity of fiber quality QTLs showing positive additive effects, conversely; however, indicates that *G. arboreum* has the underlying genes of enhancing fiber quality of *G. hirsutum*. This study provides new insights into the breeding genetic potential of *G. arboreum*, lays the foundation for further mining favorable genes of interest, and provides guidance for inter-ploidy gene transference from relatives into cultivated crops.

## Introduction

Cotton, as a textile industry crop, is of global economic importance. *Gossypium hirsutum* (2*n*=4*x*=52, AD), upland cotton, as a major cultivated tetraploid species, accounts for ~95% of cotton production worldwide (Chen et al., 2007). Numerous studies showed that there was a low level of genetic differentiation in *G. hirsutum* and that selection was extremely weak during modern genetic improvement (Fang et al., 2017a; Wang et al., 2017a), demonstrating the breadth of genetic diversity in upland cotton is narrow and the improvement of modern cotton varieties is relatively slow. Therefore, how to effectively broaden the genetic diversity of upland cotton and improve varieties to meet the increasing demands of the textile industry is the main challenge faced by researchers and breeders.

One effective strategy for broadening the genetic diversity is to transfer favorable genes into modern cultivars by intraspecific or interspecific hybridization. At present, studies are mainly focused on intraspecific *G. hirsutum* and interspecific *G. hirsutum* × *G. barbadense* populations (Said et al., 2015; Wang et al., 2019a; Zhang et al., 2019), and some elite lines have been developed for gene-function studies and breeding (Cao et al., 2014; Wan et al., 2016; Fang et al., 2017b; Feng et al., 2019; Wang et al., 2019a; He et al., 2021). However, to obtain cotton varieties with high yield, quality, and resistance, relying on these above populations is insufficient. The genus *Gossypium* possesses abundant germplasm resources, including 45 diploid (2*n*=2*x*=26) with eight genomes (A to G and K) and seven allotetraploid (2*n*=4*x*=52) species with the AD genome (Fryxell, 1992; Percival and Wendel, 1999; Wang et al., 2018). These resources provide an abundant gene pool for the improvement of upland cotton. In particular, the diploid cotton species with the largest number in *Gossypium* genus would potentially be exploited in cotton improvement programs. Several important traits of diploid species have been successfully transferred into upland cotton via specialized breeding approaches, such as cytoplasmic male sterility trait of *G. harknessii* (2*n*=2*x*=26, D_2−2_) (Meyer, 1973), low-gossypol and high-gossypol plant traits of *G. sturtianum* (2*n*=2*x*=26, C_1_) (Benbouza et al., 2010), resistance to reniform nematode (*Rotylenchulus reniformis*) of *G. aridum* (2*n*=2*x*=26, D4) and *G. longicalyx* (2*n*=2*x*=26, F) (Romano et al., 2009; Bell et al., 2014), and high fiber quality traits and immunity to bacterial blight of *G. anomalum* (2*n*=2*x*=26, B1) (Qian et al., 1992; Zhou et al., 2003). However, most of the elite genes/traits in diploid cotton remain untapped.

*Gossypium arboreum* (2*n*=2*x*=26, A2), Asian cotton species, is an Old World cultivated diploid species native to Asia. With the rapid development of spinning technology, the species was replaced by the New World allotetraploid *G. hirsutum* (Ma et al., 2008). *G. arboreum* possesses numerous invaluable characteristics unavailable in upland cotton varieties, such as resistance to pests (*Apolygus lucorum*) and diseases (caused by *Verticillium dahliae, Fusarium oxysporum vasinfectum*, and cotton leaf curl virus), high drought tolerance, and high fiber strength (Gill and Bajaj, 1987; Mehetre et al., 2003; Chen et al., 2015). Transferring these elite traits will be of great significance to the genetic improvement of *G. hirsutum*. However, a major barrier, that is, cross-incompatibility between the two species strongly hinders the transference of favorable genes. Researchers had been attempting to overcome hybridization barriers through different approaches. Sacks and Robinson (2009) crossed *G. arboreum* with a hexaploid 2[(AD_1_)D_4_] bridging the line to obtain a tetraploid triple-species hybrid, and achieved introgression of resistance to reniform nematode (*Rotylenchulus reniformis*) into upland cotton from *G. arboreum* by consecutive backcrossing. He et al. (2021) analyzed 3,248 tetraploid cotton genomes; introgression and association analyses identified new fiber quality-related loci and demonstrated that introgressed alleles on chromosome A09 from *G. arboreum* had a large effect on fiber quality, with an improvement of nearly 15% in fiber length and strength. Obviously, the transfer of elite traits from *G. arboreum* to *G. hirsutum* could make significant contributions to the genetic improvement of cotton. To date, however, numerous potentially valuable genes hidden in *G. arboreum* are still not unlocked due to the species isolation, it is, therefore, necessary to unfasten desirable genes at the whole genome level from *G. arboreum* into *G. hirsutum* via genome-wide introgression.

*Gossypium arboreum* was considered to be A_t_ subgenome donor relative of *G. hirsutum* (Stephens, 1944; Li et al., 2014; Du et al., 2018; Huang et al., 2020). Study on genomic and genetic divergence between *G. arboreum* and *G. hirsutum* has been the focus of phylogenetic studies in cotton. Gerstel (1953) first discovered there were two and three pairs of chromosomes that were translocated between *G. hirsutum* and *G. arboreum*, respectively, through hybridization. Endrizzi and Brown (1962) isolated translocation line with a four-body ring from a triplet hybrid [(*G. arboreum* × *G. herbaceum*) × *G. hirsutum*]. The cytological test and genetic studies confirmed there were reciprocal translocations between chromosomes 1, 2, 3, 4, and 5 of *G. arboreum* and *G. hirsutum*. In recent years, with the completion of genome sequencing of cotton, the evolutionary relationship A-genome between *G. hirsutum* and *G. arboreum* has been more clearly presented (Huang et al., 2020). Modern studies on genomics have shown that large chromosome structural variations exist between *G. hirsutum* and *G. arboreum*. Translocations were found between Chr01 and A03, Chr02 and A01, Chr03 and A02, Chr04 and A05, and Chr05 and A04, large inversions were found between Chr02 and A02, Chr04 and A04, Chr10 and A10, Chr11 and A11, and Chr12 and A12 (A_t_ was denoted by A + chromosome number; and A_2_ by Chr + chromosome number) (Hu et al., 2019; Shen et al., 2019; Huang et al., 2020). The chromosomal structural variation is not only related to the evolution of cotton genus but also related to some important agronomic traits. Therefore, it is of great significance to understand the genomic structural variation among the different species of cotton.

In the previous study, we overcame the cross-incompatibility between *G. arboreum* and *G. hirsutum* through the improved embryo rescue technique; successfully obtained hybrid F_1_ (2n = 39, ADA_2_) and chromosome-doubled into an amphiploid (2n = 78, AADDA_2_A_2_) (Chen et al., 2015). The synthetic amphiploid provides the foundation for genome-wide introgression from *G. arboreum* into *G. hirsutum*. The objectives of this study were (1) to develop the first set of *G. arboreum* genome-wide chromosome segment introgression lines (ILs) in *G. hirsutum* background; (2) to identify and analyze introgression at the genome-wide level, and elucidate influence of chromosome structure differentiation on introgression; (3) to map QTLs of yield-related and fiber quality traits, and reveal the potential of *G. arboreum* in improving *G. hirsutum*.

## Materials and Methods

### Plant Materials

In our previous studies, a synthetic amphiploid (AADDA_2_A_2_) was successfully obtained as deriving from an interspecific hybrid of *G. hirsutum* acc. TM-1 and *G. arboreum* cv. SXY 1 (Chen et al., 2015), and grown at the Pailou Plant Experiment Station, Nanjing Agricultural University, China in 2014. From the summer of 2014, the amphiploid plants were backcrossed as female with *G. hirsutum* acc. TM-1. The obtained hybrid seeds were planted and produced the BC_1_ population composed of 105 individuals at Nanjing in the summer of 2015, which was backcrossed again with TM-1 to generate BC_2_ seeds. Because of the sterility of certain BC_1_ individual plants, hybridization was performed again between amphiploid and TM-1 to obtain new BC_1_ seeds in the same year. Then, BC_1_ and BC_2_ populations grown at Sanya, Hainan Province in the winter of 2015, were then backcrossed with TM-1 to produce their BC_2_ and BC_3_ population, and the obtained backcross-seeds were planted at Nanjing in the summer of 2016. Finally, the produced BC_3_ and BC_4_ populations grown and were self-fertilized at Sanya in the winter of 2016. The obtained selfed seeds sowed to produce BC_3_F_2_ and BC_4_F_2_ population consisted of 236 individuals and 1,999 individuals, respectively, at Dangtu, Anhui Province in the summer of 2017. In this generation, a total of 289 BC_3_/BC_4_F_2_ individuals were retained by marker-assisted selection (MAS), and continuously self-fertilized two times to produce the BC_3_/BC_4_F_2−4_ lines (Supplementary Figure 1).

### Development of Polymorphism SSR and InDel Markers

Simple sequence repeats (SSRs) were searched on *G. hirsutum* and *G. arboreum* genomes (Zhang et al., 2015; Du et al., 2018) using MISA (http://pgrc.ipk-gatersleben.de/misa/). The microsatellite motifs were searched by the following criteria: eighteen repeat units for mononucleotide (Mono) repeats, nine for dinucleotide (Di) repeats, six for trinucleotide (Tri) repeats, four for tetranucleotide (Tetra) repeats, three for pentanucleotide (Penta) repeats, and three for hexanucleotide (Hexa) repeats. For insertion–deletions (InDels), the sequence reads of *G. arboreum* (Du et al., 2018) were prepared and aligned to the genome of *G. hirsutum* (Zhang et al., 2015) using BWA software, then InDels calling was performed with the Genome Analysis Toolkit (GATK, version v3.1) (McKenna et al., 2010), and InDels with differences ≥10 bp were retained. All primer pairs were designed in the 300-bp region on the flanking of SSR or InDel using Primer 3.0. The major parameters for designing PCR primers were as follows: (1) primer length ranging from 18 to 27 bases; (2) PCR product size ranging from 100 to 300 bp; (3) melting temperature between 55 and 65°C, with 60°C being the optimum annealing temperature; and (4) a GC content of 45–65%, with an optimum of 50%. The program e-PCR (http://www.ncbi.nlm.nih.gov/projects/e-pcr/) was utilized for simulating PCR amplification and testing specificity and product polymorphism of primers in the genomes of *G. hirsutum* and *G. arboreum*. Based on the results of the e-PCR, we finally retained SSR- and InDel-site-specific primers with the difference ≥10 bp between *G. hirsutum* and *G. arboreum*. All markers obtained with the difference ≥10 bp between *G. arboreum* and *G. hirsutum* were used to perform collinearity analysis. The sequences of the mapped markers were compared using BLAST (e-value cut-off of 1e-05) against the *G. hirsutum* and *G. arboreum* genomes (Du et al., 2018; Hu et al., 2019) to obtain the orthologous map positions of the top hits in the A_2_ genome of *G. arboreum* and At-subgenomes of *G. hirsutum*, and generated the figures using TBtools (Chen et al., 2020).

### DNA Extraction and Marker Genotyping

Total genomic DNA was extracted from young leaves using a modified cetyl trimethylammonium bromide (CTAB) method (Paterson et al., 1993). The PCR amplifications were performed using a programmable thermal controller (MJ Research). For genotyping ILs, PCR amplicon was separated by running the polypropanamide gel electrophoresis and silver staining were conducted as described by Zhang et al. (2002). All distinctive and unambiguous polymorphic bands were used for scoring and genotyping. The detected loci were named with the primer name.

To detect the introgressed chromosome segments from *G. arboreum* into *G. hirsutum*, we developed and synthesized 401 primer pairs with unambiguously polymorphic products, which could evenly distribute the At-subgenome. Based on these markers, the two-step strategy was conducted for molecular identification. First, the physical framework map consisting of 181 markers was used for a whole genome survey in BC_3_F_2_/BC_4_F_2_ generation, the purpose was to make a selection from the BC_3_F_2_/BC_4_F_2_ population through molecular marker assistance, and then 289 BC_3_F_2_/BC_4_F_2_ individuals were retained according to the distribution of introgressed loci and agronomic trait difference significantly from TM-1. Second, a high-density physical map including all 401 polymorphic markers was used to identify 289 ILs in BC_3_/BC_4_F_2−4_ generation, the purpose was to determine the homozygosity and lengths of the introgressed chromosome segments. We found there were a few heterozygous segments in BC_3_/BC_4_F_2−4_ generation, accounting for 18.90%. Then, self-crossing to BC_3_/BC_4_F_2:6_ generation, the heterozygous segments were identified by the molecular marker, and finally, all introgressed segments were homozygous. The allele from *G. arboreum* was denoted as A, whereas the allele from *G. hirsutum* was denoted as B.

### The Estimation of Introgressed Chromosome Segments

If both adjacent markers are from the donor (DD), the flanked segment was considered to be 100% donor type; if both adjacent markers are from the recipient (RR), the flanked segment was considered to be 0% donor type, and a chromosome segment flanked by one marker from the donor and another marker from the recipient (DR) was considered a 50% donor type. Therefore, the length of DD plus that of 1/2 DR was the estimated length of an introgressed chromosome segment (Xi et al., 2006). Based on the results of marker genotyping for ILs, Graphical Geno-Type2.0 software (Van Berloo, 2008) was used to analyze the introgression segments from *G. arboreum* into *G. hirsutum*. The numbers and lengths of the introgression segments were calculated using Microsoft Office Excel.

### The Measurement of Yield-Related and Fiber Quality Traits

In the summer of 2018 and 2019, 289 ILs were planted in Dangtu, Anhui province (E1) and Shangqiu, Henan province (E2), respectively, based on a randomized complete block design with two replications. Then a randomized complete block design with three replicates was applied, 289 ILs were planted in Sanya of Hainan province (2019 winter) (E3), Shangqiu of Henan province (2020 summer) (E4), and Shihezi of Xinjiang province (2020 summer) (E5). For field experiments under five environments, the recipient TM-1 and donor SXY 1 were used as control. Twenty-five bolls from each ILs in the middle of each row were hand-harvested from the internal middle parts of the plants. The yield-related traits, i.e., boll weight (BW), lint percent (LP), seed index (SI), number of bolls per plant (BN), number of fruit branches (FBN), and plant height (PH) were tested. All fiber samples from the five different environments were ginned by a roller. The fiber qualities were evaluated by high volume instrument for 2.5% fiber length (FL, mm), fiber strength (FS, cN/tex), micronaire (MIC), fiber elongation (FE, %), and fiber uniformity (FU, %). Basic statistical parameters, correlation coefficients, and phenotypic variation were performed using Microsoft Excel and SPSS 20.0 (SPSS, Chicago, IL, USA). The heritability of all the traits was calculated using QTL IciMapping 4.2 software.

### Quantitative Trait Locus Mapping and Genome-Wide Association Analysis

A likelihood ratio test based on stepwise regression (RSTEP-LRT) was used to detect the QTLs of non-idealized ILs (Wang et al., 2007, 2012). The QTL IciMapping 4.2 (http://www.isbreeding.net) was used to measure the effects of the QTLs of non-idealized ILs. A likelihood of odds (LOD) threshold of 2.5 was used to determine significant additive QTLs (Wang et al., 2006).

In order to obtain reliable QTL, we continued to conduct an association analysis between marker and phenotypic traits by a mixed linear model (MLM). STRUCTURE version 5.0 software package was used to evaluate the ILs population structure and determine the appropriate K value (Supplementary Figure 2) (Pritchard et al., 2000; Evanno et al., 2005). Then, a Q-value matrix was calculated. Kinship was observed among ILs using TSAAEL 5.0 package. Based on the Q + K + MLM model, genome-wide association analysis was carried out using TASSEL 5.0 package (Yu et al., 2006). Marker loci detected by both methods were called common QTL (co-QTL), considered to be more reliable. The co-QTL was named as follows: q + trait abbreviation + chromosome number + QTL number (Mccouch et al., 1997).

### Function Annotation of Candidate Genes in QTL Cluster

A QTL cluster is defined as a densely populated QTL region on a chromosome that contains multiple QTLs associated with various traits (Said et al., 2015). In this study, we defined a 10 Mb physical region harboring three or more QTLs as a cluster (Keerio et al., 2018). The intervals of the QTL cluster were the flanking markers of all QTLs in this region. The candidate genes were identified by the physical positions of the QTL clusters in the *G. arboreum* reference genomes (Du et al., 2018; Hu et al., 2019). Gene ontology (GO) and Kyoto encyclopedia of genes and genomes (KEGG) analyses were implemented using TBTools (Chen et al., 2020).

## Results

### Polymorphic Markers-Based Physical Map Construction

Based on the reference genomes, we searched 93,726 and 93,734 SSRs in *G. hirsutum* (Zhang et al., 2015) and *G. arboreum* (Du et al., 2018), and developed 93,708 and 93,108 pairs of SSR primers, respectively. Through e-PCR, only the site-specific primer pairs with the product difference ≥10 bp between parents were retained and finally, 535 pairs of SSR primers were obtained. For InDels, there were a total of 160,304 InDels called through aligned sequence reads of *G. arboreum* into the genome of *G. hirsutum*, most of which were in the At-subgenome (156,564 InDels). Considering the limitation of the resolution of electrophoresis, we only retained InDels located at the At-subgenome with the difference ≥10 bp, and there were 7,739 InDels obtained. Based on these InDels, a total of 7,257 pairs of primers were developed, then e-PCR was conducted to test the specificities of primer pairs and the sizes of products, and finally, 3,955 site-specific primer pairs with the product difference ≥10 bp between parents were obtained (Supplementary Table 1).

We synthesized 416 pairs of primers developed according to the physical position of all the markers, of which 401 pairs could produce unambiguously polymorphic bands with an effective rate of 96.40%, including 335 InDel and 66 SSR markers. The total spanning length of the map was 1,396.37 Mb, covering 98.17% of the At-subgenome of *G. hirsutum* (Hu et al., 2019), and the average distance was 3.55 Mb between the adjacent markers. There were 40 markers on Chr. A08, representing the most markers among the 13 chromosomes. The chromosomes with the fewest markers were Chrs. A02 and A04, with 23 markers each. The marker densities varied between chromosomes, ranging from 3.02 Mb (A07) to 4.71 Mb (A02) per marker (Supplementary Table 2 and Supplementary Figure 3). These markers were available and almost evenly distributed on the At-subgenome, which could represent the whole At-subgenome, and could be used for introgression analysis from *G. arboreum* into *G. hirsutum*.

### Introgression Analysis

**Figure 1:**
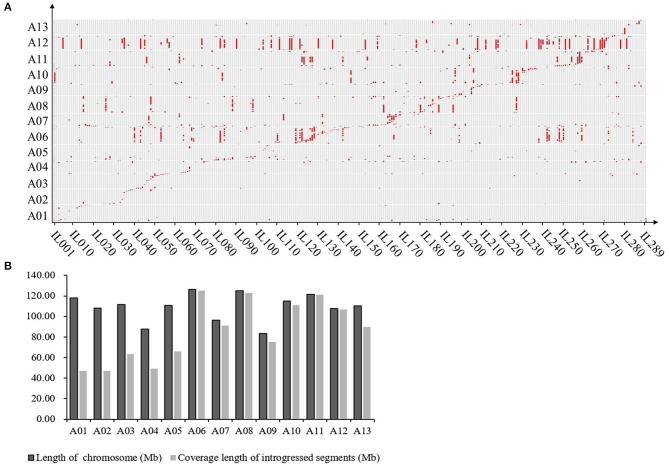
Introgression distribution for *G. arboreum* in A_t_ subgenome of *G. hirsutum*. **(A)** Graphic of genotypes of the 289 ILs. Gray regions represent *G. hirsutum* background; Red regions represent introgression segments from *G. arboretum*, and the horizontal axis represents ILs (289 in total); **(B)** Genome coverage of introgressed chromosome segments from the donor in A_t_ subgenome of *G. hirsutum*.

The results of the introgression analysis indicated the introgressed segments derived from *G. arboreum* in *G. hirsutum* background were different (Figure 1A). The total length of all introgressed segments was 9,898.93 Mb. The total length of introgressed segments on each chromosome was different, the shortest was 85 Mb on Chr. A02, while the largest was 3,229.07 Mb on Chr. A12. The minimum number of segments was on Chr. A02 with 17, and the maximum was on Chr. A06 with 179. The length of each segment varied from 0.23 Mb (Chr. A03) to 94.96 Mb (Chr. A06), with an average length of 8.69 Mb. The average length of each segment on Chrs. A01–A05 (4.35–5.87 Mb) was smaller than that on Chrs. A06–A13 (5.68–23.92 Mb) (Table 1). The total coverage length of the introgressed segments was 1,116.29 Mb, covering 78.48% of the A_t_-subgenome in *G. hirsutum* background (Figure 1B). The highest coverage rate was on Chr. A11 (99.71%) and the lowest on Chr. A01 (39.83%). The coverage rates on Chrs. A06–A13 were all >80%, being much higher than that of Chrs. A01–A05 (Table 1).

**Table 1:** Basic information of introgressed chromosome segments in the *G. hirsutum* background.

**Chr**.	**Length of chromosome (Mb)**	**Length of introgressed segments (Mb)^**a**^**	**Range of introgressed segments (Mb)**	**Number of introgressed segment**	**Mean length (Mb)**	**Coverage length of introgressed segments (Mb)^**b**^**	**Percentage of coverage (%)^**c**^**
A01	118.17	130.40	1.49–11.50	30	4.35	47.07	39.83
A02	108.27	85.00	0.94–10.60	17	5.00	47.07	43.48
A03	111.59	198.67	0.23–18.43	42	4.73	63.54	56.94
A04	87.7	201.67	1.92–14.67	41	4.92	49.11	56.00
A05	110.85	340.46	1.06–17.21	58	5.87	66.18	59.70
A06	126.49	1,781.16	0.93–94.96	179	9.95	124.84	98.69
A07	96.6	653.79	0.61–36.70	126	5.19	91.14	94.35
A08	125.06	1,002.17	1.08–59.05	80	12.53	123.02	98.37
A09	83.22	198.70	1.09–18.17	35	5.68	75.28	90.46
A10	115.1	853.43	0.59–45.21	70	12.19	111.03	96.47
A11	121.38	1,028.70	0.64–92.13	98	10.50	121.03	99.71
A12	107.59	3,229.07	0.84–84.87	135	23.92	106.93	99.38
A13	110.37	195.71	0.85–44.17	24	8.15	90.05	81.60
Total	1,422.37	9,898.93	0.23–94.96	935	8.69	1,116.29	78.48

a *The total length of all introgressed segments distributed on the chromosomes including all overlapped segments;*

b *The total length of all introgressed segments distributed on chromosomes, while the overlapped segments were calculated only once;*

c *Percentage of coverage= Coverage length of introgressed segments/Length of chromosome*.

### The Comparative Analysis of the Introgression Segments in the Structural Variation Regions of the Chromosome

**Figure 2:**
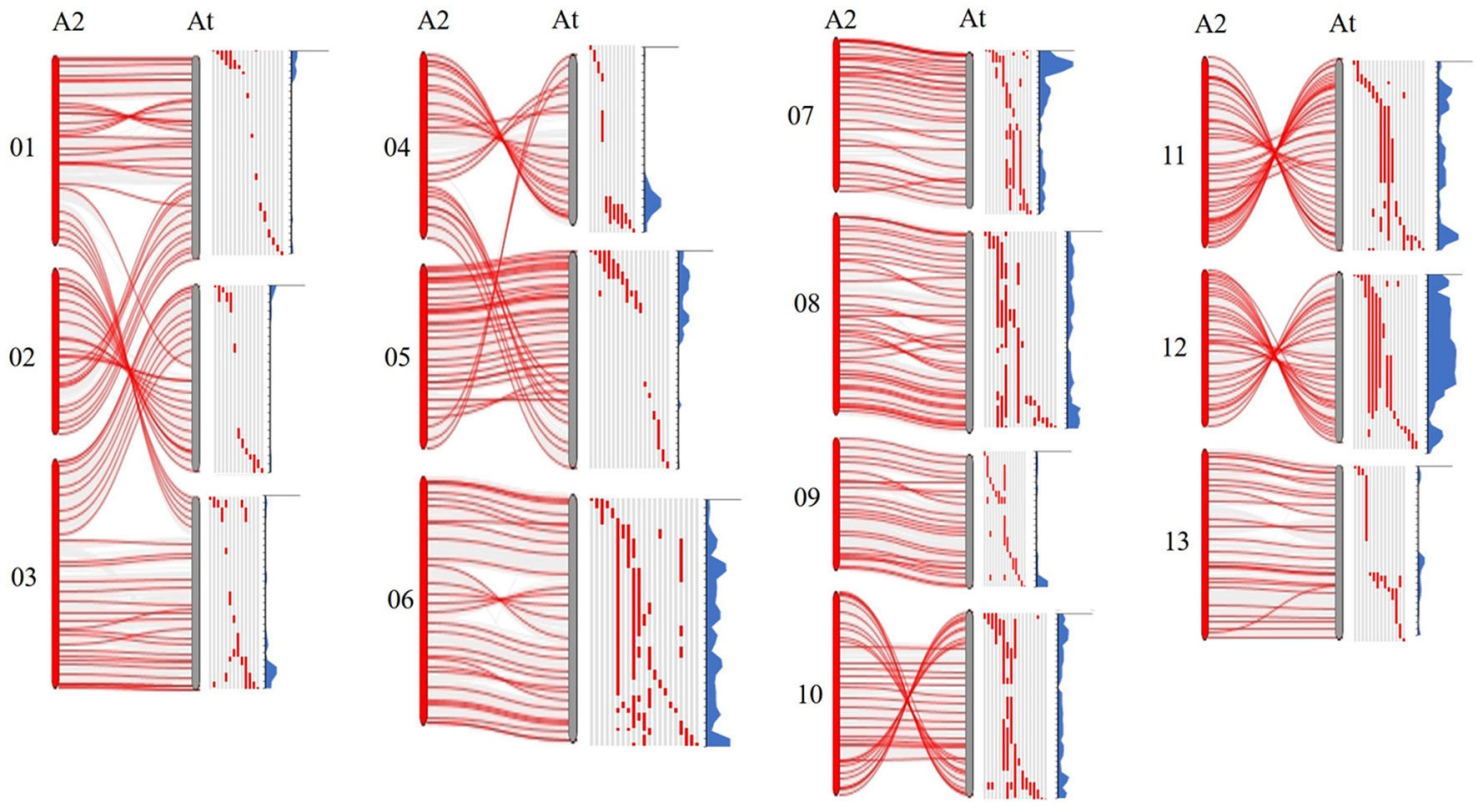
The chromosomal variant between subgenomics and distribution of introgressed segments in the A_t_ subgenome of *G. hirsutum*. A_2_ represents *G. arboreum*; A_t_ represents the A subgenome of *G. hirsutum*. Gray regions represent the *G. hirsutum* background; Red regions represent the introgression segments from *G. arboreum*. Blue parts represent the introgression rate of each locus from *G. arboreum* into *G. hirsutum*.

As previously reported, there are large chromosome structural variations that existed between the A_2_ and the A_t_-subgenomes, which is confirmed by comparative analysis using polymorphic markers developed in this study. Translocations mainly exist between Chr01 and A03, Chr02 and A01, Chr03 and A02, Chr04 and A05, and Chr05 and A04; large inversions were observed between Chr02 and A02, Chr04 and A04, Chr10 and A10, Chr11 and A11, and Chr12 and A12 (Gerstel, 1953; Hu et al., 2019; Shen et al., 2019; Huang et al., 2020). We found that distribution of *G. arboreum* introgression segments in *G. hirsutum* background showed a great difference among each chromosome. Obviously, there was a relatively lower introgression coverage rate on Chrs. A01–A05 (39.83–59.70%) (Table 1; Figure 1B) and their most introgressed segments were mainly on the distal chromosomal regions with short segments (Figures 1A, 2). Moreover, the average introgression rate was very low on each chromosome of Chrs. A01–A05, varying from 0.54 to 1.60% (Supplementary Table 3; Figure 2). Therefore, it was extremely difficult for introgression from these chromosomes of *G. arboreum* into those of *G. hirsutum*, demonstrating that reciprocal translocations involving Chrs. A01, A02, A03, A04, and A05 strongly hindered introgression. Inversely, there were higher coverage rates (81.60–99.71%) on Chrs. A06–A13 (Table 1; Figure 1B), and large-segment introgressions could be generally found on these chromosomes, especially, the largest segment was on Chrs. A06 was 94.96 Mb, accounting for 75.07% of chromosome, the largest segment A11 was 92.13 Mb, accounting for 75.90%, and the largest segment on A12 was 84.87 Mb, accounting for 78.88% (Table 1; Figure 1A). The mean introgression rates were much higher on each chromosome of Chrs. A06–A13, varying from 1.04 to 10.55% (Supplementary Table 3; Figure 2). These results demonstrated inversions between chromosomes easily suppressed chromosomal exchange and led to large-segment substitutions. It was never reported before that chromosomal structural differentiation had huge effects on genome-wide introgression in cotton.

### The Phenotypic Variation of Yield-Related and Fiber Quality Traits

There were great differences in the morphological phenotypes of *G. hirsutum* acc. TM-1 and *G. arboreum* cv. SXY 1. TM-1 exhibited cream-color petals, white anthers, palmate leaves, and big bolls, while SXY 1 showed white petals with red spots, yellow anthers, long stigmas, sub-okra leaves, and small conical bolls.

We found that there was great variation for morphological phenotype in ILs due to the introgression of *G. arboreum* (Supplementary Figure 4), such as size and color of the petals with/out red spots, size and shape of the bracts, leaves, and cotton bolls. Of them, IL051 showed white spotted petals while IL141 showed spotted yellow petals. IL129 has the smallest bract while IL022 has the largest. Especially, IL011 and IL197 captured a large fragment (~80 Mb) in Chr. A12, and showed yellow anthers and small long bolls. IL068 and IL069 contained a large fragment (~60 Mb) in Chr. A08, with small deep-lobe leaves. The descriptive statistics for the yield-related traits of the ILs along with their parents in five environments were presented in Supplementary Table 4. There were significant differences between parents in all environments except the fruit branch number (FBN) and the FE rate. TM-1 was significantly superior to SXY1 in single BW, SI, LP, FL, FS, and FU but not in PH, BN, and MIC, showing a huge evolutionary difference between *G. hirsutum* and *G. arboreum*. The transgressive segregation occurred for all 11 traits in IL population. Some ILs were even better than the recipient TM-1, suggesting the diploid *G. arboreum* had the potential to improve the yield and quality of the tetraploid *G. hirsutum*. The values of the 11 traits showed a large range of variation. For six yield-related traits, LP showed the lowest in coefficient of variation (CV) (7.77–8.84%) while BN was much higher (15.76–22.09%) than others, implying BN was easily affected by environmental factors. Of the five fiber quality traits, FU had the smallest variation (0.92–1.73%), being the most stable trait, while MIC had the largest variation (9.00–10.60%). In addition, FE in CV (E5, 23.62%) was much higher than that in the other four environments (1.49–1.92%), indicating that E5 environment has a great influence on FE.

ANOVA revealed that variances of ILs, environments, and ILs by environmental interaction were significant at the *p* = 0.01 level (Table 2). The estimated broad-sense heritability ($h_{b}^{2}$) for all traits ranged from 52.31 to 92.61%. Three yield-related traits (LP, BW, and SI) had higher $h_{b}^{2}$ while the other three traits (PH, PBN, and BN) had lower $h_{b}^{2}$ than those of five fiber quality traits. For yield-related traits, BN had the lowest $h_{b}^{2}$ (52.31%) while LP had the highest (92.61%), implying LP was much more stable than BN. For fiber quality traits, they had similar $h_{b}^{2}$ except for MIC which had slightly lower $h_{b}^{2}$ (70.32%).

**Table 2:** ANOVA and heritability for yield-related and fiber quality traits in ILs in cross-environments.

**Traits**	***F*** **test**			**Heritability (*h^**2**^*)**
	**Genotypes**	**Environments**	**Genotypes × Environments**	
BW	22.98^**^	2,028.58^**^	2.168^**^	88.44
SI	17.81^**^	1,283.59^**^	2.498^**^	87.6
LP	67.29^**^	4,240.81^**^	1.464^**^	92.61
PH	3.13^**^	3,136.25^**^	2.269^**^	64.98
FBN	2.47^**^	2,117.11^**^	2.266^**^	64.8
BN	2.24^**^	3,104.72^**^	4.603^**^	52.31
FL	16.48^**^	714.06^**^	2.364^**^	82.47
FS	9.47^**^	162.68^**^	2.032^**^	81.78
MIC	19.9^**^	1,327.57^**^	2.18^**^	70.32
FU	2.99^**^	1,665.6^**^	1.291^**^	84.84
FE	6.85^**^	576.67^**^	4.732^**^	78.08

*BW, boll weight; SI, seed index; LP, lint percent; PH, plant height; FBN, fruit branch number; BN, boll number; FL, fiber length; FS, fiber strength; MIC, micronaire; FU, fiber uniformity; FE, fiber elongation. E1, Dangtu, Anhui province in 2018; E2, Shangqiu, Henan province in 2019; E3, Sanya, Hainan province in 2019; E4, Shangqiu, Henan province in 2020; E5, Shihezi, Xinjiang province in 2020. Significance level:*

** *p < 0.01*.

The correlation analysis showed that significant positive correlations were observed between the trait pairs of BW-SI, BW-FL, BW-FS, SI-FL, SI-FS, LP-MIC, PH-FBN, PH-BN, FBN-BN, FL-FS, and FL-FE; and significant negative correlations were observed between the pairs of BW-LP, SI-LP, LP-FL, LP-FS, FL-MIC, and FS-MIC (Supplementary Table 5).

Fortunately, out of 289 ILs, it was observed that yield components (single BW, LP, and BN) and fiber quality traits (fiber length, strength, and micronaire) were simultaneously improved in the four ILs, implying that negative correlations between yield component traits and quality traits can be broken (Table 3).

**Table 3:** The yield components (single BW, LP, and BN) and fiber quality traits (FL, FS, and MIC) were simultaneously improved in the four ILs.

**ILs**	**BW (g)**	***P*-value**	**SI (g)**	***P*-value**	**LP (%)**	***P*-value**	**PH (cm)**	***P*-value**	**PBN**	***P*-value**	**BN**	***P-*value**
NB032	6.98 ± 1.40	0.11	14.36 ± 1.74	0.09	36.07 ± 3.59^*^	0.04	105.46 ± 26.02	0.33	13.26 ± 3.10	0.42	21.90 ± 11.32	0.35
NB137	6.26 ± 1.13	0.45	13.85 ± 1.59	0.19	34.34 ± 3.25	0.17	104.38 ± 26.37	0.36	12.49 ± 2.94	0.42	23.85 ± 14.86	0.28
NB186	6.73 ± 0.92	0.12	13.32 ± 1.20	0.36	34.75 ± 3.22	0.12	105.81 ± 23.16	0.31	11.93 ± 2.51	0.29	20.07 ± 8.63	0.47
NB279	6.38 ± 1.03	0.35	13.92 ± 1.39	0.15	34.80 ± 2.36	0.08	93.32 ± 19.55	0.34	12.00 ± 2.66	0.31	19.90 ± 8.64	0.48
TM-1	6.19 ± 0.52		13.03 ± 1.46		32.56 ± 2.83		98.93 ± 24.78		12.87 ± 3.17		19.69 ± 8.40	
**ILs**	**FL (mm)**	***P*** **-value**	**FS (cN/tex)**	***P*** **-value**	**MIC**	***P*** **-value**	**FU (%)**	***P*** **-value**	**FE**	***P-*** **value**		
NB032	30.23 ± 1.83	0.17	31.90 ± 2.13^**^	0.00	4.08 ± 0.31^*^	0.02	84.55 ± 2.36	0.46	6.28 ± 0.80	0.38		
NB137	29.31 ± 0.92	0.44	28.80 ± 2.57	0.37	4.25 ± 0.31^*^	0.05	82.92 ± 1.90	0.10	6.53 ± 0.37	0.33		
NB186	29.40 ± 2.21	0.44	30.37 ± 1.36^**^	0.01	4.43 ± 0.36	0.13	85.11 ± 2.17	0.29	6.36 ± 0.61	0.45		
NB279	29.42 ± 1.22	0.40	30.65 ± 2.26^*^	0.04	4.25 ± 0.29^*^	0.04	85.65 ± 2.11	0.17	6.16 ± 0.99	0.31		
TM-1	29.21 ± 1.22		28.38 ± 0.90		4.77 ± 0.52		84.43 ± 1.56		6.41 ± 0.45			

*BW, boll weight; SI, seed index; LP, lint percent; PH, plant height; FBN, fruit branch number; BN, boll number; FL, fiber length; FS, fiber strength; MIC, micronaire; FU, fiber uniformity; FE, fiber elongation*.

*Significance level:*

* *p < 0.05*,

** *p < 0.01*.

### Quantitative Trait Locus Mapping and Genome-Wide Association Study (GWAS) for Yield-Related and Fiber Quality Traits

A total of 159 QTLs were detected for 11 traits based on QTL mapping of RSTEP-LRT analysis, including 91 for yield and 68 for fiber quality, explaining 1.17–24.78% of the phenotypic variation, with an average of 4.55%. The distribution of QTLs on each chromosome was different. For yield traits, most QTLs were detected on A08 (17), while only one QTL was detected on A01. For fiber quality traits, most QTLs (12) were detected on A06, while no QTL for fiber quality was detected on A01 and A13 (Supplementary Table 6).

In this study, a total of 185 marker loci were identified, by the GWAS analysis method, to be associated with yield or fiber quality traits (Supplementary Table 7). Their PVEs ranged from 1.01 to 7.15%, with an average of 2.82%. Likewise, it was different for the distribution of associated loci on each chromosome. For yield traits, most loci were associated on A08 (18), while only one marker was associated with A01. For fiber quality traits, most loci (15) were associated on A08, while only one marker was associated with A01 and A13.

### Common QTLs Detected for Yield-Related and Fiber Quality Traits

**Figure 3:**
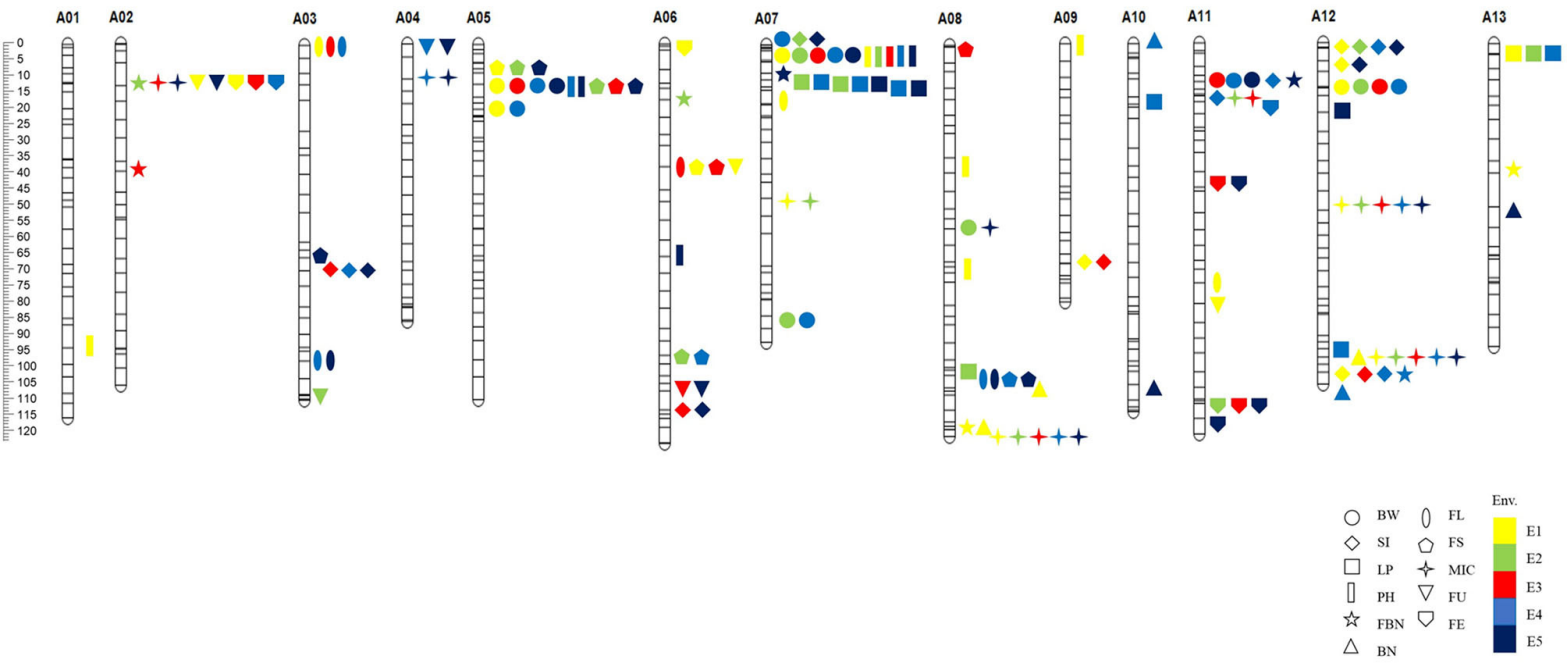
The distribution of co-QTLs for yield-related and fiber quality traits on chromosomes. BW, boll weight; SI, seed index; LP, lint percent; PH, plant height; FBN, fruit branch number; BN, boll number; FL, fiber length; FS, fiber strength; MIC, micronaire; FU, fiber uniformity; FE, fiber elongation. E1, Dangtu, Anhui province in 2018; E2, Shangqiu, Henan province in 2019; E3, Sanya, Hainan province in 2019; E4, Shangqiu, Henan province in 2020; E5, Shihezi, Xinjiang province in 2020.

Marker loci detected by both methods were called co-QTLs, which were more reliable in this study. There were a total of 81 co-QTLs that were detected on the At subgenome in the *G. hirsutum* background and 47 QTLs for yield and 34 for fiber quality (Figure 3). The position, LOD/P-score, additive effects, and percentages of phenotypic variance explained (PVE) of the QTLs were given in Supplementary Table 8. These QTLs explained 1.01–24.78% of PVE with an average of 3.76%. Twenty-three QTLs were detected in multiple environments by both methods, called stable QTLs.

#### Boll Weight

There were seven co-QTLs for BW detected on four chromosomes with PVE ranging from 1.17 to 11.56%. Chrs. A11 and A12 each had one QTL, Chrs. A05 and A07 had two and three QTLs, respectively. Among them, six QTLs showed negative additive effects while only qBW-A07-3 showed a positive additive effect, suggesting that introgression of *G. arboreum* mainly decreased the phenotypic value for BW in the *G. hirsutum* background. Moreover, qBW-A05-1, qBW-A05-2, qBW-A07-2, qBW-A11-1, and qBW-A12-1 could be detected in multiple environments by both methods.

#### Seed Index

Nine co-QTLs for SI were detected in total, their PVE ranged from 1.53 to 8.55%. Chrs. A03, A06, A07, A09, each had one QTL; Chr. A11 had two QTLs; Chr. A12 had three QTLs. Five QTLs (qSI-A07-1, qSI-A11-1, qSI-A11-2, qSI-A12-1, and qSI-A12-2) showed negative additive effects; and four QTLs (qSI-A03-1, qSI-A06-1, qSI-A09-1, and qSI-A12-3) showed positive additive effects. qSI-A03-1, qSI-A06-1, qSI-A12-1 were detected in multiple environments by both methods.

#### Lint Percent

Eight co-QTLs conferring LP were detected on five chromosomes and their PVEs ranged from 1.21 to 10.79%. Chrs. A08, A10, and A13 each had one QTL, Chrs. A12 and A07 had two and three QTLs, respectively. Among them, qLP-A10-1, qLP-A12-1, qLP-A12-2, and qLP-A13-1 showed positive additive effects, while qLP-A07-1, qLP-A07-2, qLP-A07-3, and qLP-A08-1presented negative additive effects. qLP-A07-2 could be detected in multiple environments by both methods.

#### Plant Height

There were seven co-QTLs for PH detected on six chromosomes with PVE ranging from 1.50 to 13.09%. Chrs. A01, A05, A06, A07, and A09 each had one QTL; Chr. A08 had two QTLs. Among them, four QTLs (qPH-A01-1, qPH-A06-1, qPH-A08-1, and qPH-A09-1) showed positive additive effects and the other three (qPH-A05-1, qPH-A07-1, and qPH-A08-2) showed negative additive effects. qPH-A07-1 were detected in multiple environments by both methods.

#### Number of Fruit Branches

Eight co-QTLs related to FBN were detected, located on Chrs. A02, A06, A07, A08, A11, A12, and A13, respectively, and their PVP ranged from 2.11% to 5.48%. Six QTLs (qFBN-A02-1, qFBN-A06-1, qFBN-A07-1, qFBN-A08-1, qFBN-A11-1, and qFBN-A13-1) showed positive additive effects while only three QTLs (qFBN-A02-2 and qFBN-A12-1) presented negative additive effects, suggesting that *G. arboreum* had the potential to improve the FBN of *G. hirsutum*.

#### Number of Bolls per Plant

There were eight co-QTLs for BN detected on four chromosomes with PVE ranging from 2.15 to 8.83%. Chr. A13 had one QTL, Chrs. A10 and A12 each had two QTLs, and Chr. A08 had three QTLs. Among them, three QTLs (qBN-A08-2, qBN-A12-1, and qBN-A12-2) presented negative additive effects, and five QTLs (qBN-A08-1, qBN-A08-3, qBN-A10-1, qBN-A10-2, and qBN-A13-1) had positive additive effects, suggesting that *G. arboreum* had potential in improving the BN of *G. hirsutum*. The result was similar to that of FBN, demonstrating *G. arboreum* was superior to *G. hirsutum* in the evolution or domestication of BN.

#### Fiber Length

Seven co-QTLs for FL were detected on five chromosomes, their PVE ranged from 1.57 to 7.53%. Chrs. A06 and A08 and A11 each had one QTL, Chrs. A03 and A07 each had two QTLs. Among them, five QTLs (qFL-A06-1, qFL-A07-1, qFL-A07-2, qFL-A08-1, and qFL-A11-1) showed negative additive effects, while only two QTLs, qFL-A03-1 and qFL-A03-2 showed a positive additive effect, suggesting that introgression of *G. arboreum* mainly decreased the phenotypic value for FL in the *G. hirsutum* background. Moreover, qFL-A03-1, qFL-A03-2, and qFL-A08-1 could be detected in multiple environments by both methods.

#### Fiber Strength

There were seven co-QTLs for FS detected on four chromosomes with PVE ranging from 1.35 to 9.71%. Chr. A03 had one QTL and Chrs. A05, A06, and A08 each had two QTLs. Among them, five QTLs (qFS-A05-1, qFS-A05-2, qFS-A06-1, qFS-A06-2, and qFS-A08-2) presented negative additive effects, and only two QTLs (qFS-A03-1 and qFS-A08-1) had positive additive effects, suggesting that introgression of *G. arboreum* mainly decreased the phenotypic value for FS in the *G. hirsutum* background. qFS-A05-1, qFS-A05-2, and qFS-A08-2 were detected in multiple environments by both methods.

#### Micronaire

Eight co-QTLs for MIC were detected on six chromosomes, their PVE ranged from 1.72 to 8.55%. Chrs. A02, A04, A07, A11, and A12 each had one QTL, Chrs. A08 had two QTLs. Among them, four QTLs (qMIC-A02-1, qMIC-A08-1, qMIC-A08-2, and qMIC-A12-1) showed positive additive effects, other four QTLs, qMIC-A04-1, qMIC-A07-1, qMIC-A08-2, and qMIC-A11-1, showed negative additive effect. qMIC-A04-1, qMIC-A08-2, qMIC-A11-1, qMIC-A12-1, and qMIC-A12-2 could be detected in multiple environments by both methods.

#### Fiber Uniformity

Six co-QTLs for FU were detected on five chromosomes with PVE ranging from 1.01 to 7.62%. Chrs. A02, A03, A04, and A11 each had one QTL, Chr. A06 had two QTLs. Among them, five QTLs (qFU-A02-1, qFU-A03-1, qFU-A06-1, qFU-A06-2, and qFU-A11-1) presented negative additive effects, and only one QTLs (qFU-A04-1) had positive additive effects, suggesting that introgression of *G. arboreum* mainly decreased the phenotypic value for FU in the *G. hirsutum* background. qFU-A06-2 was detected in multiple environments by both methods.

#### Fiber Elongation

There were six co-QTLs for FE detected on three chromosomes with PVE ranging from 1.21 to 24.78%. Chrs. A02 and A06 each had one QTL and Chr. A11 had four QTLs. Among them, four QTLs (qFE-A02-1, qFE-A06-1, qFE-A11-3, and qFE-A11-4) presented negative additive effects, and only two QTLs (qFE-A11-1 and qFE-A11-2) had positive additive effects, suggesting that introgression of *G. arboreum* mainly decreased the phenotypic value for FE in the *G. hirsutum* background. qFE-A02-1 was detected in multiple environments by both methods.

### Common-QTL Cluster Identification

Among the co-QTLs for yield and fiber quality, there were multiple co-QTLs for different traits located on the same intervals, which were considered as co-QTL clusters. In this study, we found eight co-QTL clusters with 38 QTLs on seven chromosomes (Table 4; Figure 3). Six of the co-QTL clusters contained at least one stable QTL, implying that key genes existed that were related to yield traits or fiber quality in these regions. Seven QTL clusters were associated with not only yield but also fiber quality, and A06-cluster-1 was associated with only fiber quality traits.

**Table 4:** The distribution of co-QTL clusters.

**Cluster name**	**Number of QTLs**	**Include QTLs**	**Approximate position in *G. hirsutum* (Mb)**
A02-cluster-1	4	qFBN-A02-1 (+)	A02: 5.76–11.17
		qMIC-A02-1 (+)	
		qFU-A02-1 (–)	
		qFE-A02-1 (–)	
A05-cluster-1	4	qFS-A05-1 (–)	A05: 6.25–11.14
		qBW-A05-1 (–)	
		qPH-A05-1 (–)	
		qFS-A05-2 (–)	
A06-cluster-1	3	qFL-A06-1 (–)	A06:37.10–43.93
		qFS-A06-1 (–)	
		qFU-A06-1 (–)	
A07-cluster-1	8	qFL-A07-1 (–)	A07:0.27–12.52
		qBW-A07-1 (–)	
		qSI-A07-1 (–)	
		qBW-A07-2 (–)	
		qPH-A07-1 (–)	
		qFBN-A07-1 (+)	
		qLP-A07-1 (–)	
		qLP-A07-2 (–)	
A08-cluster-1	4	qLP-A08-1 (–)	A08:100.49–108.46
		qFL-A08-1 (–)	
		qFS-A08-2 (–)	
		qBN-A08-2 (–)	
A08-cluster-2	3	qFBN-A08-1 (+)	A08:120.33–124.01
		qBN-A08-3 (+)	
		qMIC-A08-2 (+)	
A11-cluster-1	6	qBW-A11-1 (–)	A11:11.54–16.85
		qSI-A11-1 (–)	
		qFBN-A11-1 (+)	
		qSI-A11-2 (–)	
		qMIC-A11-1 (–)	
		qFE-A11-1 (+)	
A12-cluster-1	6	qLP-A12-2 (+)	A12: 95.35–105.60
		qBN-A12-1 (–)	
		qMIC-A12-2 (–)	
		qSI-A12-3 (+)	
		qFBN-A12-1 (–)	
		qBN-A12-2 (–)	

*BW, boll weight; SI, seed index; LP, lint percent; PH, plant height; FBN, fruit branch number; BN, boll number; FL, fiber length; FS, fiber strength; MIC, micronaire; FU, fiber uniformity; FE, fiber elongation; (+) positive and (–) negative additive effect QTLs. Gh represents physical region in G. hirsutum background; Ga represents physical region deriving from G. arboreum*.

There were four QTLs observed in the A02-cluster-1 at the approximate position of 5.76–11.17 Mb on Chr. A02 in *G. hirsutum*, qFBN-A02-1 and qMIC-A02-1 had positive additive effects while qFU-A02-1 and qFE-A02-1 showed negative additive effects. A05-cluster-1 contained four QTLs at the approximate position of 6.25–11.14 Mb on Chr. A05 in *G. hirsutum*, all QTLs showed negative additive effects. The A06-cluster-1 contained three QTLs at the approximate position of 37.10–43.93 Mb on Chr. A06 in *G. hirsutum*, and all QTLs related to fiber quality and showed negative additive effects. There were eight QTLs observed in A07-cluster-1, where the approximate position was 0.27–12.52 Mb on Chr. A08 in *G. hirsutum*, all QTLs showed negative additive effects except qFBN-A07-1. The A08-cluster-1 contained four QTLs at the approximate position of 100.49–108.46 Mb on Chr. A08 in *G. hirsutum*, and all QTLs showed negative additive effects. The A08-cluster-2 contained three QTLs at the approximate position of 120.33–124.01 Mb on Chr. A08 in *G. hirsutum*, and all QTLs showed positive additive effects, including two QTLs related to yield (qFBN-A08-1 and qBN-A08-3), which could be used to improve BN of *G. hirsutum*. There were eight QTLs observed in the A11-cluster-1, where the approximate position was 11.54–16.85 Mb on Chr. A11 in *G. hirsutum*, four QTLs (qBW-A11-1, qSI-A11-1, qSI-A11-2, and qMIC-A11-1) showed negative additive effects, and two QTLs (qFBN-A11-1 and qFE-A11-1) had positive additive effects. There were eight QTLs in A12-cluster-1 at the approximate position 95.35–105.60 Mb on Chr. A12 in *G. hirsutum*, likewise, four QTLs (qBN-A12-1, qMIC-A12-2, qFBN-A12-,1 and qBN-A12-2) showed negative additive effects, and two QTLs (qLP-A12-2 and qSI-A12-3) showed positive additive effects.

### Function Annotation of Genes in Co-QTL Clusters

In eight co-QTL clusters, the introgressed genes were collected according to the position of the QTL cluster in the *G. arboreum* genome. There were a total of 2,726 genes introgressed from *G. arboreum* (Table 5). The A07-cluster-1 cluster harbored the most genes (922), while the A12-cluster-1 harbored 61 genes. To predict the functions of the genes, each was annotated with GO and KEGG, of which 1,905 genes had annotation information (Supplementary Tables 9, 10). There were classified into three main types containing molecular function, biological process, and cellular components through GO annotation (Supplementary Figure 5). In the molecular function category, most genes were related to ATP binding and protein binding, playing important regulatory roles in cellular activity. In the biological process category, most genes were enriched in the regulation of transcription, DNA-templated protein phosphorylation, and oxidation-reduction process. In the cellular component category, genes were mainly enriched in the integral component of membrane and membrane. The KEGG analysis indicated that eight QTL clusters were mainly involved in ribosome and signal transduction of plant hormones, amino sugar and nucleotide sugar metabolism, and phenylpropanoid biosynthesis pathways (Supplementary Figure 6).

**Table 5:** The distribution of candidate genes from *G. arboreum* in eight co-QTL clusters.

**Cluster name**	**Chromosome**	**Start**	**End**	**Number of genes**
A02-cluster-1	Chr03	6,605,679	12,808,248	277
A05-cluster-1	Chr05	6,350,714	11,311,998	541
A06-cluster-1	Chr06	38,118,856	45,523,866	94
A07-cluster-1	Chr07	325,044	12,924,878	922
A08-cluster-1	Chr08	101,817,612	110,433,764	268
A08-cluster-2	Chr08	124,179,049	128,114,083	363
A11-cluster-1	Chr11	110,057,011	106,894,935	200
A12-cluster-1	Chr12	10,523,063	5,236,512	61

## Discussion

### Polymorphic Markers Provide the Basis for Marker-Assisted Breeding and Gene Function Research

Polymorphic markers have practical implications in cotton marker-assisted breeding and map-based gene cloning (Liu et al., 2015). The SSR and InDel markers are characterized by their high frequency, wide distribution, co-dominance, and high polymorphism. With the release of high-quality cotton genome reference sequences, SSRs and InDels with polymorphism are allowed to be developed on a genome-wide level. Numerous SSR and InDel markers had been developed previously from genomic or EST sequences in cotton (Guo et al., 2007; Hinchliffe et al., 2011; Lu et al., 2015; Wang et al., 2017b), however, they were used mainly on intra-ploidy populations. The development of polymorphic markers was seldom reported to be used earlier on inter-ploidy populations. In this study, e-PCR was used to test the specificity and polymorphism of primers between *G. hirsutum* and *G. arboreum*, and we finally found 3955 InDel- and 535 SSR-site-specific primer pairs with the product difference ≥10 bp between parents. The strategy of e-PCR can not only virtually simulate the PCR process but can also save time and reduce laboratory costs. Here, we synthesized 416 primers pairs based on physical distribution, of which 401 pairs could produce unambiguously polymorphic bands with an effective rate of 96.40%, showing this strategy was reliable. Notably, we found InDels had more abundant variation between parents due to the number of InDels was much bigger than that of SSRs. These genome-wide polymorphic primer pairs undoubtedly have practical implications in cotton marker-assisted breeding and map-based cotton gene cloning. Finally, the obtained 401 available markers covered 98.17% of the At-subgenome of *G. hirsutum* with 3.55 Mb average distance between adjacent markers and were evenly distributed across the At-subgenome, providing reliable tools for introgression identification in this study (Supplementary Table 2 and Supplementary Figure 3). Meanwhile, these markers developed will lay the genetic foundation for elucidating the molecular mechanism that forms the differences in the quality, yield, and resistance to biotic/abiotic stress between *G. hirsutum* and *G. arboreum*.

### Genome-Wide Introgression From *G. arboreum* Into *G. hirsutum* Represented an Important Step for Genetic Research on Cotton

*G. arboreum* contains numerous valuable characteristics unavailable in upland cotton varieties, which are not being used effectively in breeding so far due to cross-incompatibility between *G. arboreum* and *G. hirsutum* (Sacks and Robinson, 2009; Chen et al., 2015). Genome-wide introgression from *G. arboreum* into *G. hirsutum* could greatly broaden the available gene pools in *G. hirsutum*. The gene pools of improved *G. hirsutum* are mainly derived from allotetraploid cotton germplasm resources, such as wild or semi-wild and *G. barbadense* species. To continuously improve the varieties of cotton in yield, quality, or tolerance to biotic and abiotic stress, it is essential to transfer the desired characters from the diploid species to the cultivated allotetraploid species (Zhai et al., 2015). Although several elite genes of *G. arboreum* had been successfully cloned and transferred into *G. hirsutum* (Sacks and Robinson, 2009), genome-wide introgression has not been completed, leading to a number of elite genes locked in *G. arboreum* being unable to be unfastened in *G. hirsutum* breeding. The A_2_ genome of *G. arboreum* is supposed to be close to the progenitor of the At-subgenome of *G. hirsutum* (Wendel et al., 2009), being very closely related to each other. Therefore, the genetic recombination between them is easier than between *G. hirsutum* and other non-A or non-D diploid species of cotton. In this research, the synthetic amphiploid (AADDA_2_A_2_) derived from (TM-1 × SXY 1) was used as a bridge to continuously backcross with TM-1, finally, we developed the first ILs of *G. arboreum* cv SXY 1 (donor) in *G. hirsutum* acc. TM-1 (recipient), covering ~78.48% of the At-subgenome (Table 1). The developed ILs broke the limitation of introgression with certain traits or genes from diploid, and offered an opportunity for the efficient utilization of *G. arboreum* in *G. hirsutum*. This study also provided a reference for introgression of other related diploid species into cultivated upland cotton.

### Chromosome Structure Differentiation Extremely Affects Genome-Wide Introgression From *G. arboreum* Into *G. hirsutum*

In this study, we also observed there were large translocations between Chr01 and A03, Chr02 and A01, Chr03 and A02, Chr04 and A05, and Chr05 and A04, and large inversions were found between Chr02 and A02, Chr04 and A04, Chr10 and A10, Chr11 and A11, and Chr12 and A12 (Figure 2), which was consistent with previous studies (Hu et al., 2019; Shen et al., 2019; Wang et al., 2019b). Shen et al. (2017) reported that recombination rates in the region of structural variation varied greatly, and the translocation regions and the inversion regions on *G. hirsutum* possessed relatively high recombination rates in the distal regions of the chromosomes. Similarly, we found that introgressions were extremely suppressed on Chrs. A01–A05 due to their translocations, the reason was that exchanges occurring on translocated chromosome often produced massive abortive gametes with deficiency-duplication chromosomes. Thus, we observed that their lower coverage rates (39.83–59.70%) and introgressed-segments mainly distributed on the distal end of the chromosome had relatively short segments. On the contrary, there was a higher coverage rate (81.6–99.71%), and large-segment introgressions could be generally found on Chrs. A06–A13. The reason was that inversions between chromosomes easily suppress chromosome exchange, leading to direct substitution of certain large genome segments (Figure 2). These results primarily showed chromosome structural variation extremely affects genome-wide introgression from *G. arboreum* into *G. hirsutum*.

### Genome-Wide Introgression Reveals the Genetic Potential of Asian Cotton to Improve Yield-Related and Fiber Quality Traits of Upland Cotton

Introgression lines possess the potential to reveal the inheritance of new alleles from donor species, to identify candidate genes, and to develop genome-wide genetic resources due to their uniform genetic background (recipient) excepting the introgression segments (donor) (Ali et al., 2010). The QTLs mapped by molecular markers could provide a better understanding of the genetic basis for yield-related and fiber quality traits of *G. arboreum* in *G. hirsutum*.

**Figure 4:**
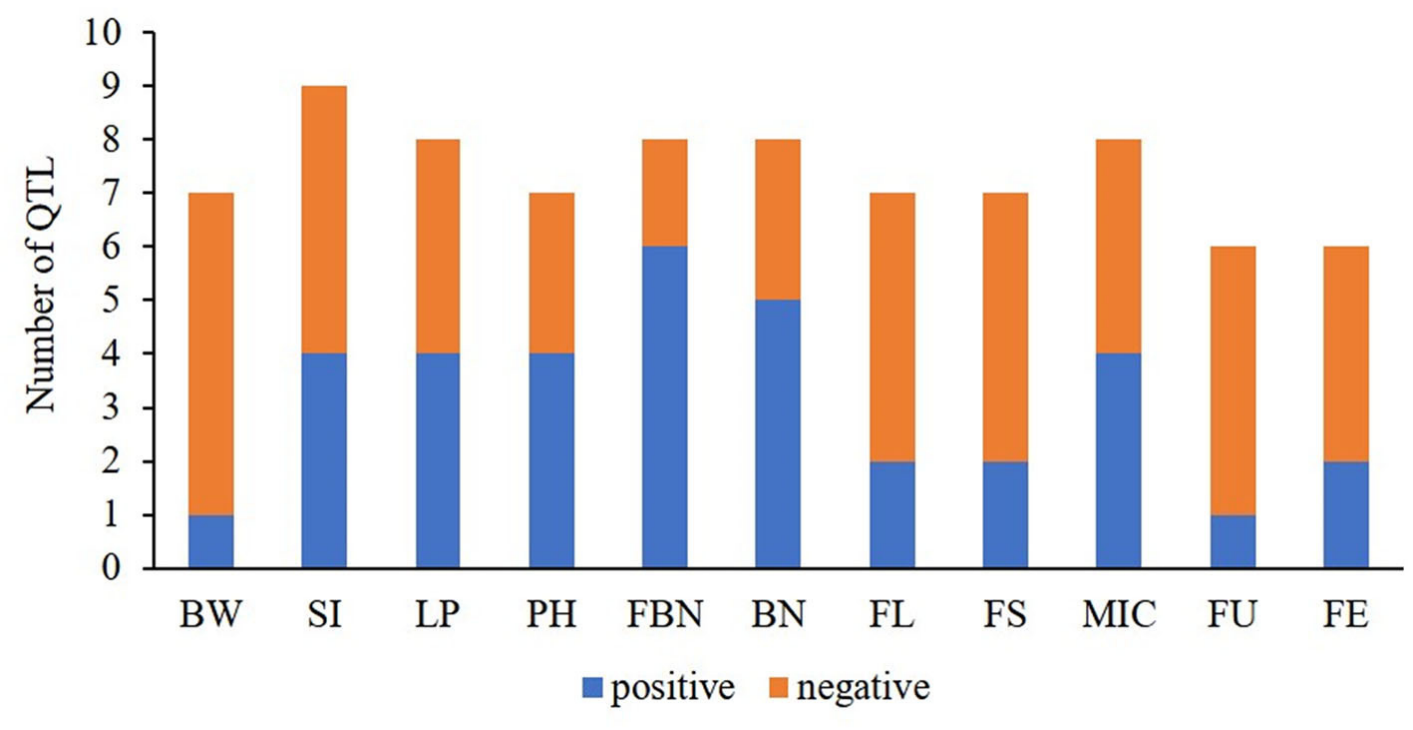
The ratios of positive- vs. negative-QTLs for yield-related and fiber quality traits. BW, boll weight; SI, seed index; LP, lint percent; PH, plant height; FBN, fruit branch number; BN, boll number; FL, fiber length; FS, fiber strength; MIC, micronaire; FU, fiber uniformity; FE, fiber elongation.

In this study, a total of 81 co-QTLs were detected (Supplementary Table 8), including 47 QTLs for yield and 34 for fiber quality. Of them, 23 stable co-QTLs were detected. In addition, we found 46 co-QTLs showed negative additive effects and 35 co-QTLs had positive additive effects from *G. arboreum* in *G. hirsutum* background. Interestingly, QTLs of yield components related to single bolls such as BW, SI, and LP mainly produced negative additive effects, while yield components related to plant such as PH, FBN, and BN mainly exhibited positive additive effects (Figure 4). The results suggested that *G. hirsutum* have produced high lint yields for a single boll, while *G. arboreum* was superior in BN-related traits for the whole plant. Therefore, *G. arboreum* could be used to improve *G. hirsutum* production by increasing BN.

Most of the QTLs related to fiber quality showed negative additive effects from *G. arboreum*, demonstrating that these QTLs had been domesticated into excellent loci in *G. hirsutum* compared to *G. arboreum* (Figure 4). However, there was a small quantity of co-QTLs showing positive additive effects. For example, qFL-A03-1 and qFL-A03-2 showed positive additive effects detected in multiple environments by both methods. Likewise, qMIC-A04-1, qMIC-A11-1, and qMIC-A12-2 showed negative additive effects and increased fiber fineness, detected in multiple environments by both methods. These results indicated that *G. arboreum* has the potential to improve fiber length and fineness.

One QTL cluster with eight co-QTLs, A08-cluster-2, contained three QTLs with positive additive effects. Of them, two QTLs were related to BN (qFBN-A08-1 and qBN-A08-3), indicating the underlying genes related to BN existed in the region.

In addition, several QTLs detected in the present research could be found in previous studies. For example, qBW-A07-3 was close to the position of the loci (Chr07: 80449650 and Chr07: 84998593) as reported by Du et al. (2018), qLP-A08-1 was close to the locus A08:103614728 as reported by Ma et al. (2018), qFS-A06-2 was close to locus A06: 95105470 as reported by Fang et al. (2017b). These QTLs consistent with earlier studies provided a solid basis for further genetic study and molecular marker breeding.

We also found several QTL clusters (Table 4; Figure 3) were at similar positions to those reported by Said et al. (2015). For example, A02-cluster-1 at the approximate position of 5.76–11.17 Mb, located at the proximal end of the chromosome A02, similar to that of c2-cluster-Gh-1: 0–20 cm; A05-cluster-1 at the approximate position of 6.25–11.14 Mb, located at the proximal end of the chromosome A05, similar to that of c5-cluster-Gh-1: 0–20 cm. A07-cluster-1 at the approximate position of 0.27–12.52 Mb, located at the proximal end of chromosome A07, similar to that of c7-cluster-Gh-1: 0–18 cm. A08-cluster-1 and A08-cluster-2 were at the approximate positions of 100.49–108.46 and 120.33–124.01 Mb, respectively. They were located at the end of chromosome A07, similar to that of c8-cluster-Gh×Gb-3: 116–137 cm. The QTLs clustered at the same position indicated the existence of principal genomic areas responsible for yield and fiber traits.

Totally, 2,726 genes introgressed from *G. arboreum* were detected in co-QTL clusters. To better understand the potential biological functions of these genes in QTL clusters, we performed GO analysis and KEGG analysis. The results showed functions of genes in QTL clusters mainly involved in signal transduction and metabolism. The current results provided the foundation for further functional studies to dissect the genetic mechanism of yield and fiber quality traits of *G. arboreum* in *G. hirsutum* background.

In summary, we have achieved genome-wide introgression from *G. arboreum* into *G. hirsutum* and determined the distribution of introgressed *G. arboreum* segments via molecular identification, which should be the important resources for desirable gene discovery and genetic analysis. We also observed that chromosome structural variation extremely affects genome-wide introgression. Our QTL mapping revealed the inheritance of yield-related and fiber quality traits of *G. arboreum* in *G. hirsutum* background, providing the possibility for improving the lint yield and the fiber quality of *G. hirsutum* using *G. arboreum*.

## Data Availability Statement

The original contributions presented in the study are included in the article/Supplementary Material, further inquiries can be directed to the corresponding author/s.

## Consent for Publication

All authors have read the manuscript and approved its publication.

## Author Contributions

BZ conceived, designed the project, revised the manuscript, and provided the research funds. LF wrote the manuscript, analyzed the morphological, and molecular marker data. YC provided the hexaploid of *Gossypium hirsutum-G. arboreum* and its BC1 progenies. LF, YY, MX, HY, QS, and CZ performed SSR marker experiments. LF, MX, HY, CZ, GF, NA, and NW performed field experiments in Dangtu, Sanya, Shangqiu, and Shihezi, respectively. MX designed SSR and Indel primers. All authors contributed to the article and approved the submitted version.

## Conflict of Interest

The authors declare that the research was conducted in the absence of any commercial or financial relationships that could be construed as a potential conflict of interest.

## Publisher's Note

All claims expressed in this article are solely those of the authors and do not necessarily represent those of their affiliated organizations, or those of the publisher, the editors and the reviewers. Any product that may be evaluated in this article, or claim that may be made by its manufacturer, is not guaranteed or endorsed by the publisher.
