# Supplementary material for: Genome-Wide Introgression and Quantitative Trait Locus Mapping Reveals the Potential of Asian Cotton (Gossypium arboreum) in Improving Upland Cotton (Gossypium hirsutum)
Source: Front Plant Sci. 2021 Aug 2;12:719371. doi: 10.3389/fpls.2021.719371 (PMC8365338; doi:10.3389/fpls.2021.719371)
Supplement: Supplementary file 1 [file Data_Sheet_1.PDF]

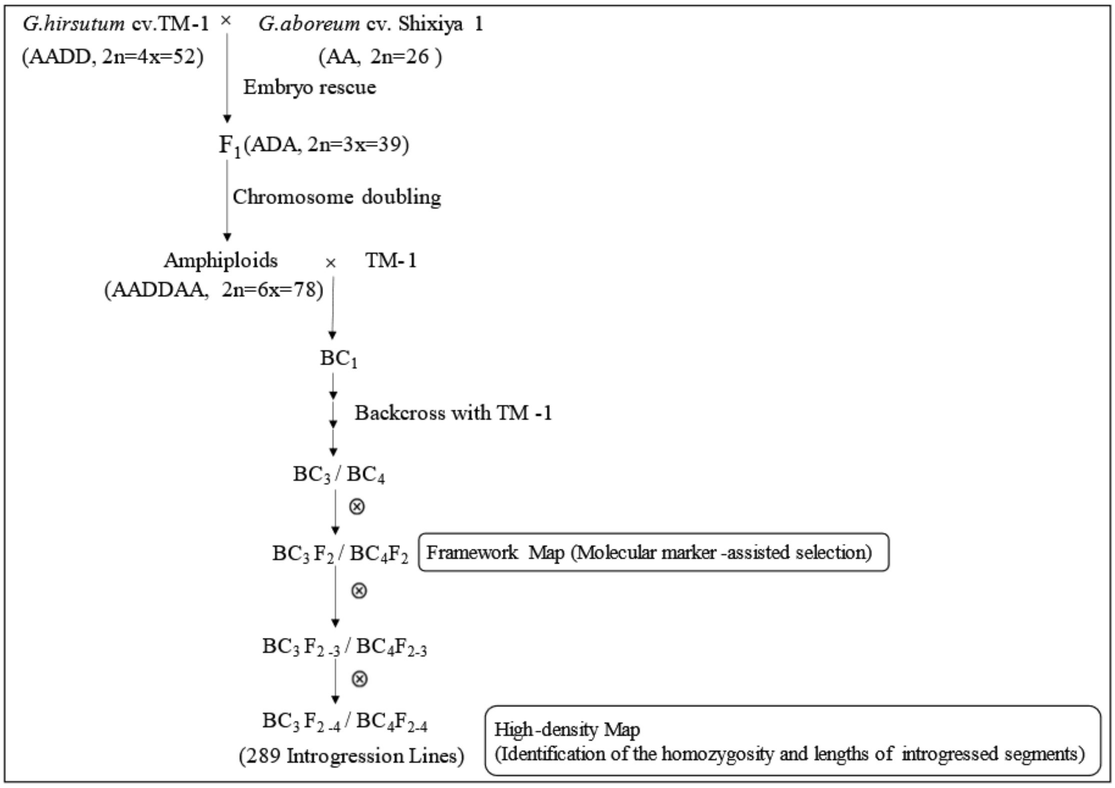

Figure S1. Development of introgression lines

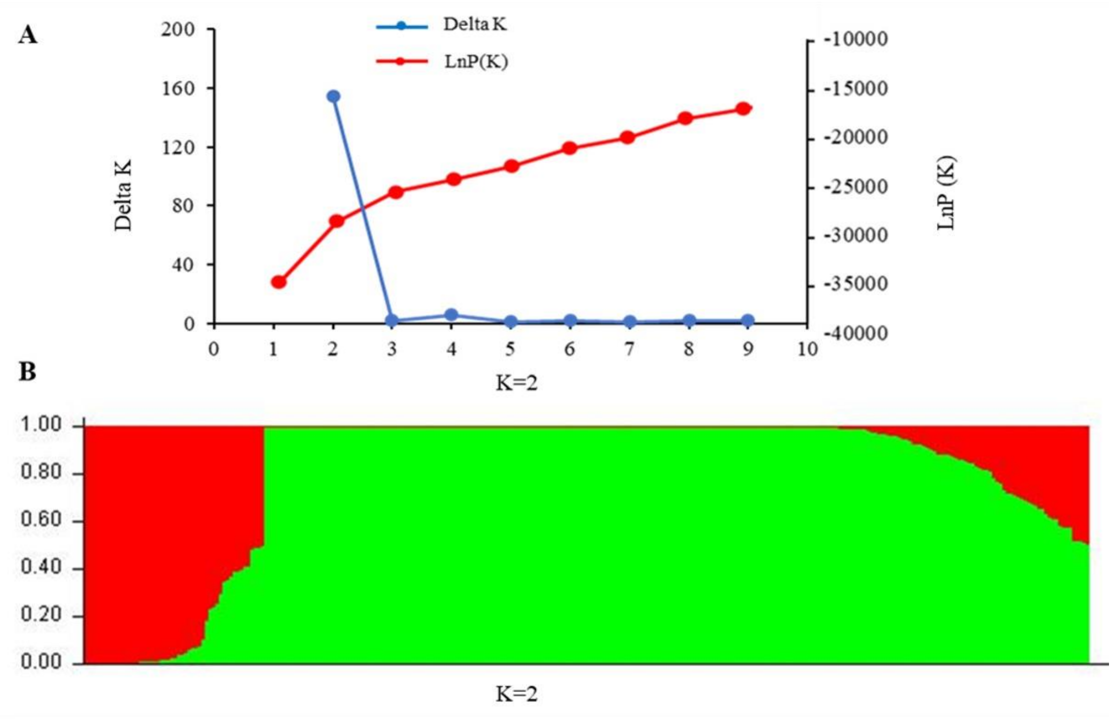

Figure S2. **Population structure of 289 ILs**. A: Plot of ΔK and LnP (K) calculated for K = 1–10. B: Population structure (K = 2), the areas of the two colors (green and red) illustrate the proportion of each subgroup.

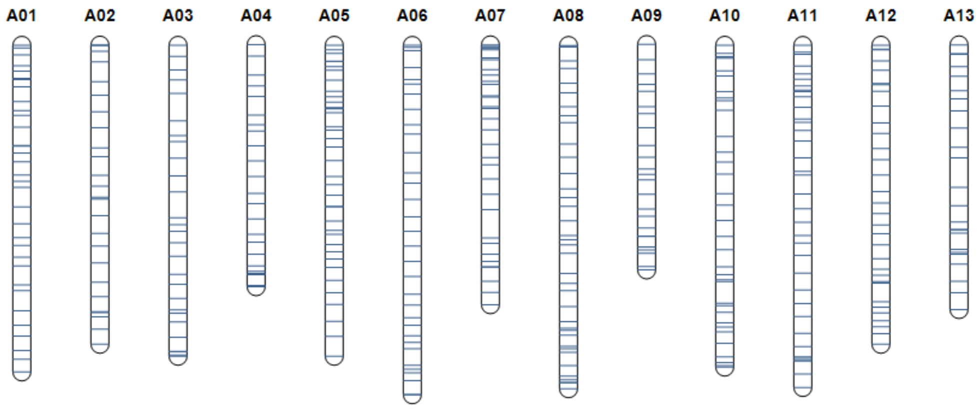

Figure S3. Distribution of 401 markers with high polymorphism in A$_t$-subgemome of *G. hirsutum*

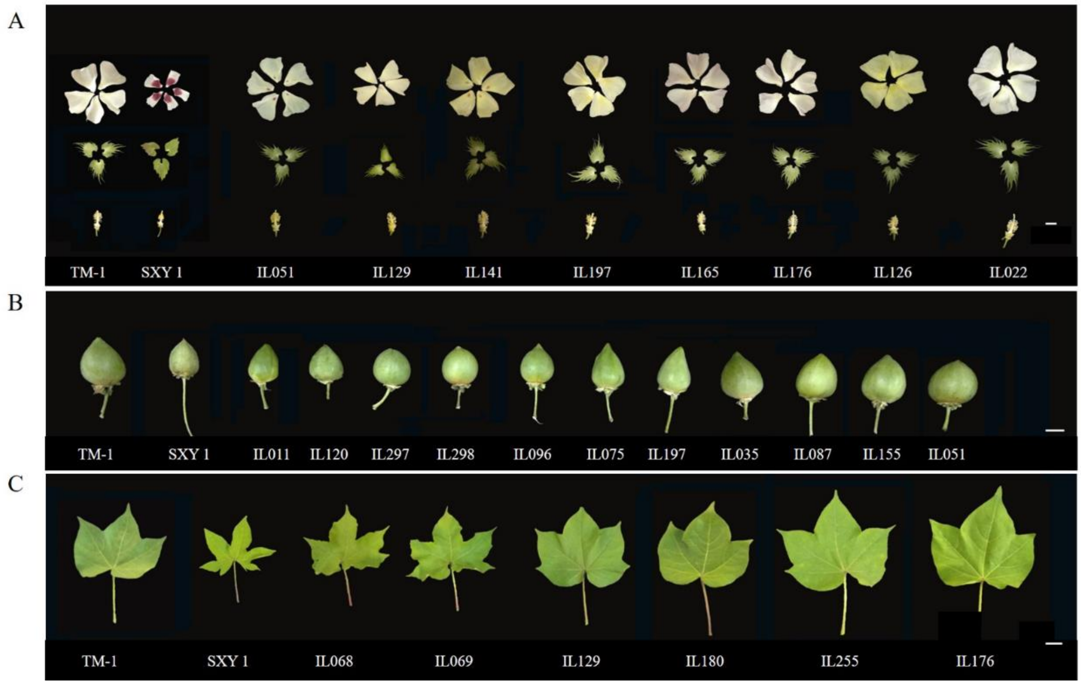

Figure S4. Morphology variances of the *G. hirsutum* TM-1, *G. arboreum* SXY 1 and ILs. A: Petals, bracts, pistils and stamens; B: Cotton bolls; C, Leaves. Scale bars, 2 cm

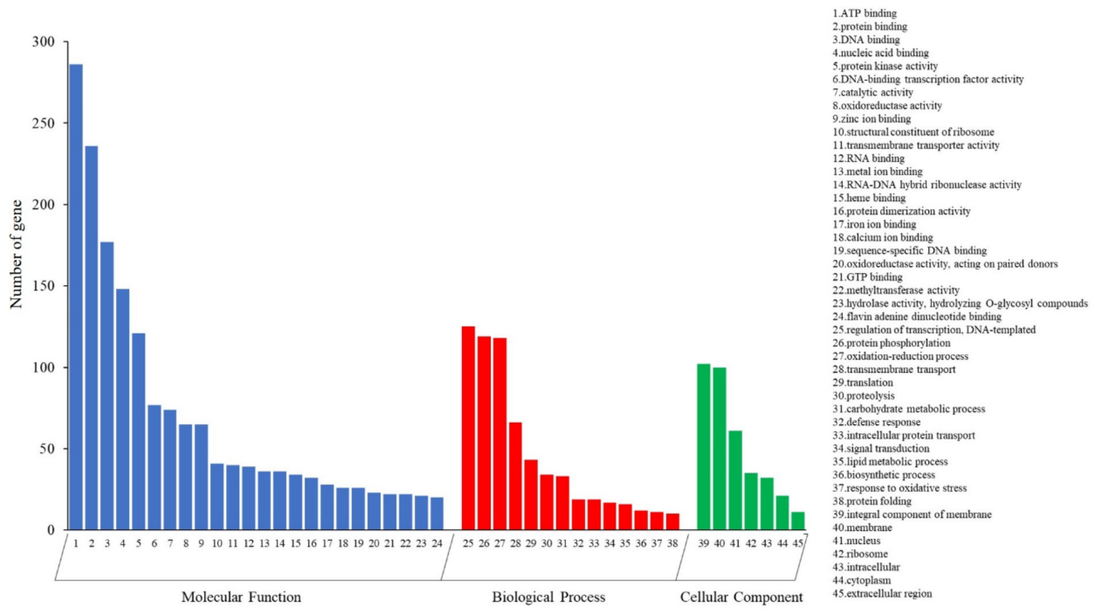

Figure S5. KEGG pathway annotation of genes introgressed from *G. arboreum* in QTL cluster region. The horizontal axis represents the KEGG classification and description; the vertical axis shows the gene numbers.

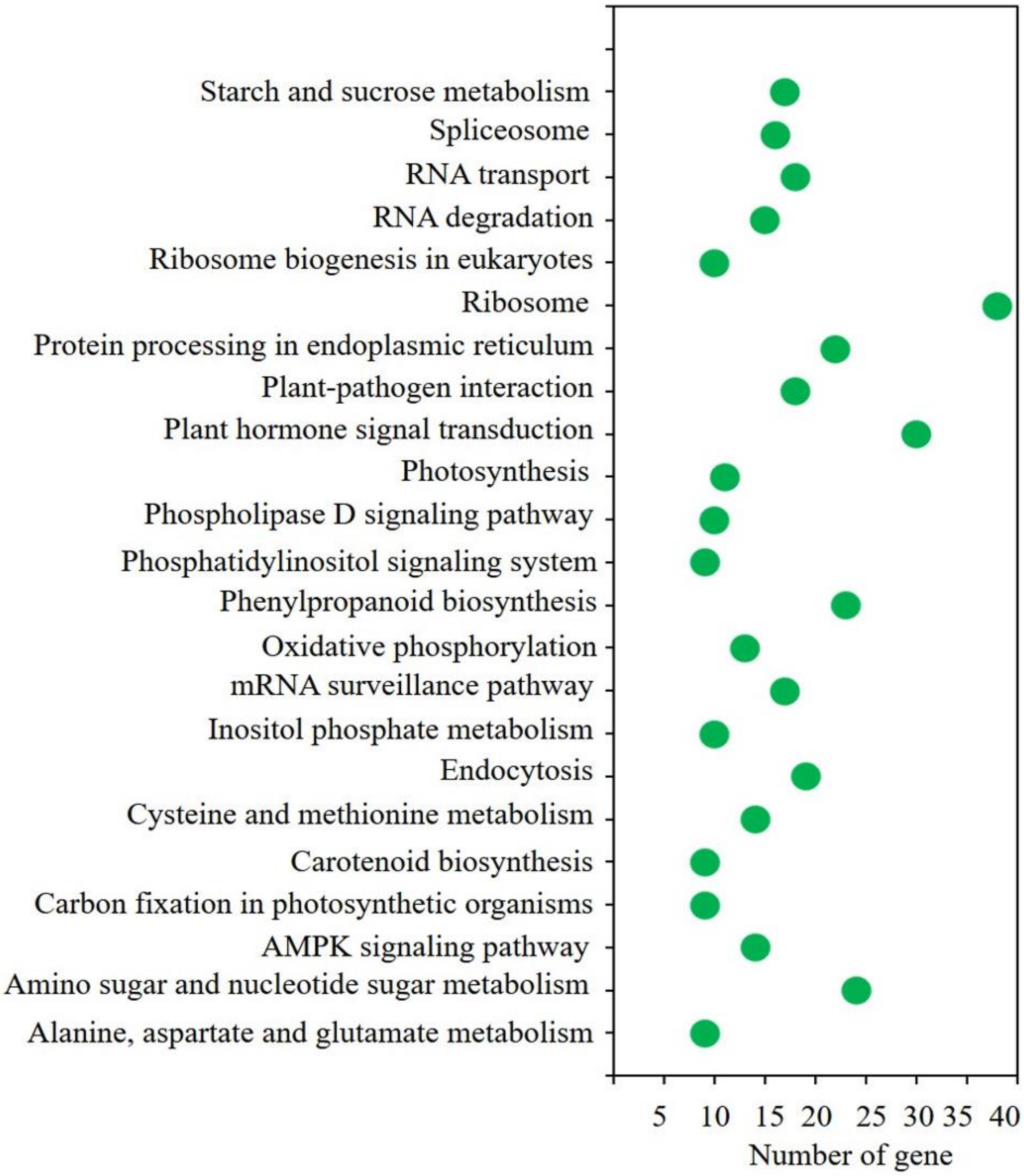

Figure S6. GO terms of genes introgressed from *G. arboreum* in QTL cluster region. The horizontal axis represents number of gene; the vertical axis shows the GO term and description.
